# Childhood socioeconomic position and physical capability in late-middle age in two birth cohorts from the Copenhagen aging and midlife biobank

**DOI:** 10.1371/journal.pone.0205019

**Published:** 2018-10-01

**Authors:** Gitte Lindved Petersen, Jolene Lee Masters Pedersen, Naja Hulvej Rod, Erik Lykke Mortensen, Ichiro Kawachi, Merete Osler, Åse Marie Hansen, Rikke Lund

**Affiliations:** 1 Section of Social Medicine, Department of Public Health, University of Copenhagen, Copenhagen, Denmark; 2 Center for Healthy Aging, University of Copenhagen, Copenhagen, Denmark; 3 Section of Epidemiology, Department of Public Health, University of Copenhagen, Copenhagen, Denmark; 4 Unit of Medical Psychology, Section of Environmental Health, Department of Public Health, University of Copenhagen, Copenhagen, Denmark; 5 Department of Social and Behavioral Sciences, Harvard School of Public Health, Boston, MA, United States of America; 6 Center for Clinical Research and Prevention, Bispebjerg and Frederiksberg Hospitals, Frederiksberg, Denmark; 7 National Research Centre for the Working Environment, Copenhagen, Denmark; TNO, NETHERLANDS

## Abstract

This study examines the association between childhood socioeconomic position and objective physical capability including new functional measures of potential relevance to a population in late-middle age. The study population covers two Danish birth cohorts followed-up in the Copenhagen Aging and Midlife Biobank (age 48–58 years, 2009–2011, N = 4,204). Results from linear regression models revealed that being born in higher socioeconomic position was associated with higher jump height: Paternal occupational class four = 0.19 cm (95% confidence interval (CI): -0.44, 0.82), three = 0.59 cm (95% CI: -0.02, 1.19), two = 1.29 cm (95% CI: 0.64, 1.94), and one = 1.29 cm (95% CI: 0.45, 2.13) (reference = five); medium parental social class = 0.88 cm (95% CI: 0.03, 1.72) and high = 1.79 cm (95% CI: 0.94, 2.63) (reference = low). Higher childhood socioeconomic position was also associated with better chair rise performance and hand grip strength, while among women it was related to reduced flexibility: Medium parental social class = -1.31 cm (95% CI: -3.05, 0.42) and high = -2.20 cm (95% CI: -3.94, -0.47) (reference = low); unwed mother = 1.75 cm (95% CI: 0.36, 3.14) (reference = married). Overall, the findings suggest that higher childhood socioeconomic position is primarily related to moderately better scores in the most strenuous physical capability measures and hand grip strength among healthy adults in late-middle age.

## Introduction

**Fig 1:**
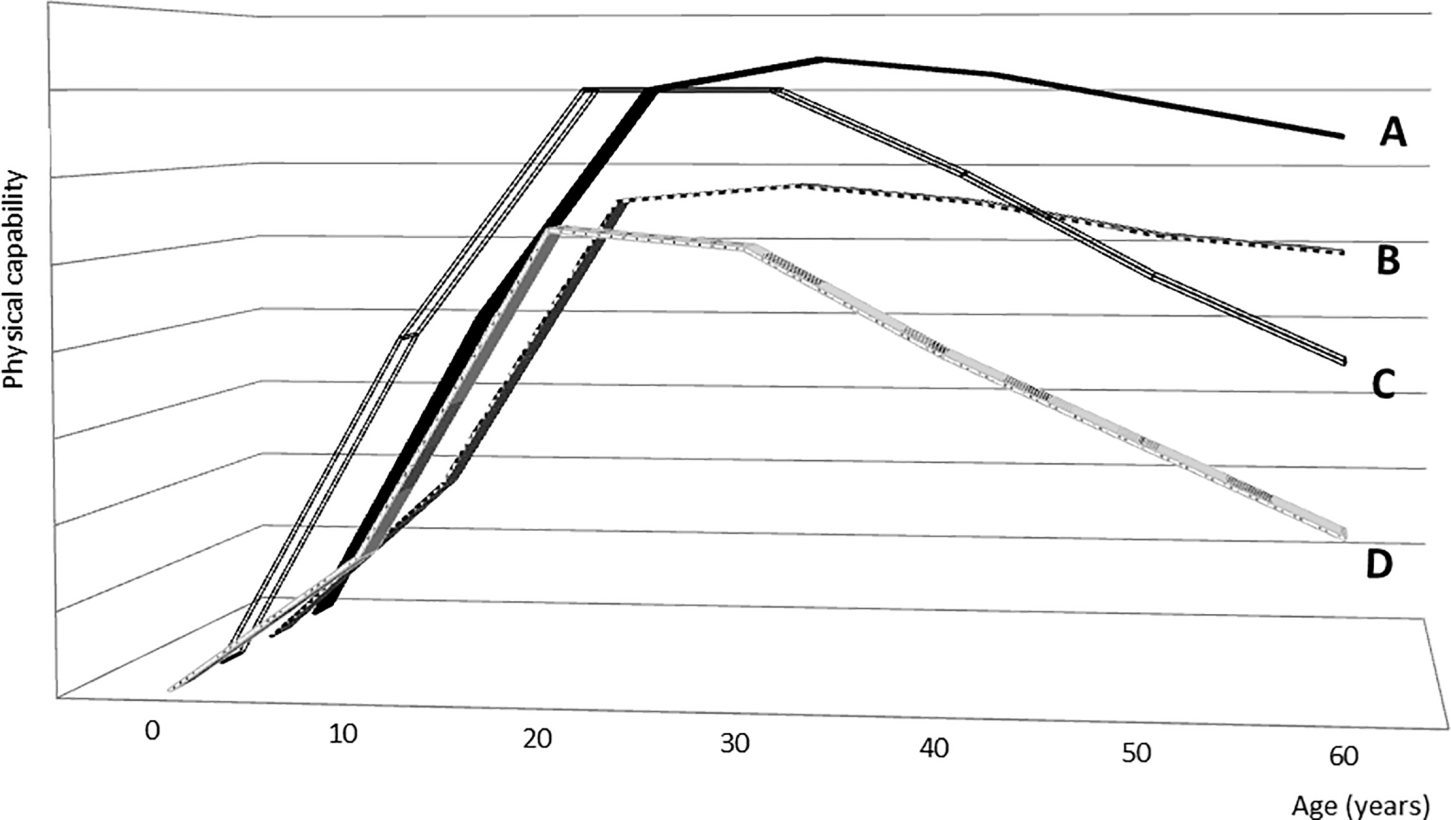
Graph illustrating examples of four functional trajectories. A: typical physiological development and decline, B: impaired physiological development but typical rate of decline, C: typical physiological development but accelerated decline, D: impaired physiological development and accelerated decline. The model is inspired by Ben-shlomo & Kuh 2002 (A life course approach to chronic disease epidemiology: conceptual models, empirical challenges and interdisciplinary perspectives. Int. J. Epidemiol. 31, 285–293. [14]) and Strachan & Sheikh 2004 (A life course approach to respiratory and allergic diseases, in: Kuh, D., Ben-Shlomo, Y. (Eds.), A Life Course Approach to Chronic Disease Epidemiology. Oxford University Press, 10, pp. 240–259. [15]).

Literature suggests that the well-established social gradient in physical capability partly originates in early life [1–12]. The relation is suggested to pass through intermediate factors such as childhood growth and development (cognitive and physiological), adult socioeconomic position (SEP), health behavior and health status [5,13]. Childhood SEP could be of particular importance because of its impact on the physiologic reserve from which age-related decline begins [14]. People who achieve higher peak levels of physical capability in early adulthood will experience functional disability at a later stage than those who were weaker at their best conditioning on the same rate of age-related decline in physical capability (paths A and B, Fig 1). Additionally, a strong physiological reserve could delay or even prevent severe functional disability in aging individuals experiencing accelerated decline (paths C and D, Fig 1).

Objectively measured physical capability is recognized as a biomarker of early aging [16] and is consistently and inversely related to all-cause mortality in older populations [17]. Numerous scientific studies utilize measures such as hand grip strength, chair rise performance and balance [16]. The chair rise performance test requires the participant to be able to lift his/her own body weight several times at a brisk pace, which is considered quite physically demanding. However, little attention has been directed towards other more vigorous tests of muscle strength (for example jump height), although such may better discriminate physical capability levels in healthy individuals in late-middle age. Further, previous tests are mostly focused on the extremities ignoring the central part of the body (i.e. back and abdomen), which may be of importance to activities of daily living.

Previous evidence has shown that higher childhood SEP is modestly but consistently related to more favorable scores on adult chair rise performance, hand grip strength, and walking speed on an absolute scale and standing balance on a relative scale with adult SEP possibly explaining some of the relationships [4–6,12]. However, considerable heterogeneity is observed across studies and it is unknown whether this represents actual differences or differences in SEP indicators and/or outcome measures [4]. Previous findings are also inconsistent in relation to potential sex interactions with some reporting no difference [1,3–5], while others do find different associations [6,7,12]. Hypothesized explanations include that women could be more vulnerable to stress (e.g. from being in low SEP) than men, and that they tend to use emotional coping strategies instead of problem-focused [12]. Early life SEP is also suggested to be more strongly related to health behaviors such as smoking and physical activity in women than men [7]. These issues are relevant to explore further because population differences could result in lack of generalizability to other populations or societies.

This study includes unexplored aspects of physical capability (jump height, lower back force, abdominal force and flexibility) using a Danish cohort. The objective is to investigate the associations between childhood SEP and physical capability in late-middle age utilizing different SEP indicators and a number of objective physical capability measures. Further, it is investigated whether the relationship exists independently of adult SEP and if it differs according to sex.

## Materials and methods

This study included members of the Metropolit Cohort (MC) and the Copenhagen Perinatal Cohort (CPC) who also participated in the in the Copenhagen Aging and Midlife Biobank (CAMB) [18]. The MC included boys born in the Copenhagen metropolitan area in 1953 [19] and the CPC included boys and girls born at the Copenhagen National University Hospital in 1959–1961 [20]. Participants from the birth cohorts who were alive and residing in eligible study areas in the Eastern part of Denmark in 2009–2011 were invited for the CAMB follow-up (N = 13,032) including comprehensive questionnaires and physical examinations [18]. In total, 4,204 men and women (32.3%) participated in the physical testing and were thus eligible for this study.

### Childhood SEP

In the MC, information on father’s occupational class was obtained in 23 strata from birth certificates and categorized into five groups: one (land proprietor; self-employed and manager with academic degree; manager; employee, civil servant with academic degree), two (factory owner; merchant, ship owner; self-employed and manager or employee, civil servant with no academic degree; other superior), three (farm owner; other self-employed in farm, fish, industry, transport, service; other employee or civil servants), four (small holder; skilled worker) and five (unskilled workers).

In the CPC, information on parental social class was obtained at the one-year follow-up on a 20-point scale including breadwinner’s education, occupation, income type, and housing quality. A score of zero-five points was given (higher score is more favorable) within each domain and the summarized score was categorized into nine categories (zero-four points = lowest social class, five-six = second, …, and 19–20 = highest social class) [20]. The nine-point measure was categorized into three groups as lower (categories one-three), middle (categories four-five), and upper (categories six-nine) class.

For all participants in CAMB, maternal marital status was used as a proxy for childhood SEP because unmarried mothers are usually more financially deprived than married mothers [21–23]. Information on maternal marital status (married, separated, divorced, widowed or single) was obtained from birth certificates in the MC and at birth in the CPC. For this study, maternal marital status was categorized as married versus unmarried.

### Physical capability

Seven aspects of physical capability were tested at CAMB follow-up (MC: age 55–58 years, CPC: age 48–52 years) [18,24,25]. Not all participants completed all tests due to exclusion criteria being more extensive for some tests than for others.

Balance was measured as one-legged postural sway area in cm^2^ during 30 seconds (n = 4,043) using an AMTI power plate [26]. The participant decided which leg to stand on and performed the test three times. The smallest sway area was used. Flexibility was measured as maximal trunk flexion with straight legs as the fingertip-to-toe distance in cm (n = 3,710). Jump height in cm was used to measure maximal muscle power in the lower extremities (n = 3,603). The test was performed as a two-legged countermovement jump on the AMTI power plate with the hands placed on the hips. The test was performed a maximum of five times and the highest score was used. Lower back force was measured in Newton (N) using a Vishay Nobel KIS-2 dynamometer (n = 3,438). The participant performed three-five maximal isometric contractions of the lower back muscles in an upright standing position pressing the hips against a plate and pulling backwards with the dynamometer attached with a strap around the shoulders. Lower back force was measured in Newton to take into account the height of the participant. The best score was used. Abdominal force was measured in N (n = 3,474) like lower back force, but facing away from the dynamometer pulling away from it in a forward bend. Grip strength was measured as max kg using a Jamar dynamometer model G100 with automatic recording (n = 4,192). The participant performed three-five attempts and the best was used. Chair rise performance was used to measure functional ability in the lower limbs as the maximum number of rises from a 45-cm high chair with automatic recording within 30 seconds (n = 3,770). The test was performed with no use of the arms.

All tests were performed at the same study clinic by trained test personnel according to detailed protocols and procedures to reduce the risk of systematic variation. Further details on the physical tests are described elsewhere [18,24].

### Covariates

The hypothesized causal relations in our study are depicted in a Directed Acyclic Graph (S1 Fig). Adult SEP was reported in late-middle age as highest attained vocational education in seven categories: none; semi-skilled worker; skilled worker or similar level (e.g. carpenter, smith, clerical training, hairdresser, nursing assistant, technical assistant); under three years theoretical education (e.g. market economist, mechanical engineer); three-four years theoretical education (e.g. primary school teacher, journalist, bachelor of engineering, bachelor degree); long further and higher education (more than four years) (e.g. doctor, economist, upper secondary school teacher, master of engineering); or other education.

### Ethical approval

Written informed consent was obtained from all participants. The Ethical Committee of the Capital Region of Denmark and the Danish Data Protection Agency have approved CAMB as a database combining three cohorts (Approval no. H-A- 2008–126 and no. 2013-41-1814). No additional approvals are required according to Danish law.

### Statistics

Characteristics of the study population are presented as frequencies with column-% and medians with inter-quartile ranges (IQR) according to birth cohort and sex.

The associations between childhood SEP and physical capability in late-middle age were tested using linear regression models. Statistical analyses were performed for the full cohort when using marital status as the exposure measure (adjusted for originating birth cohort), while analyses were conducted separately for each birth cohort when using father’s occupational class (MC) or parental social class (CPC) as exposure measures, since these were not available for both cohorts. Statistical model assumptions were tested using fit diagnostics and probability plots. The outcome variable for balance was transformed using the natural logarithm to approach normality and back-transformed for presentation resulting in interpretation as a relative change [27].

Sensitivity analyses were performed to assess the robustness of our findings. First, analyses including maternal marital status as the exposure were performed stratified by birth cohort and with a test for additive interaction. Second, we stratified analyses according to year of birth to test for potential cohort effects. Third, we were concerned about potential selective dropout because individuals of low socioeconomic position were less likely to participate in the Copenhagen Aging and Midlife Biobank (CAMB) follow-up and because exclusion criteria prevented participants in poor health to participate in a number of the physical capability tests [18]. To evaluate the potential impact of this selective drop out, we conducted a simple quantitative bias analysis, following the methods described by Lash *et al*. [28].

Sub-analyses were conducted to further explore the findings. First, potential sex differences were investigated in stratified sub-analyses and tests for interaction were performed by including an exposure-sex product term in the models. Second, analyses were additionally adjusted for the intermediate factor adult SEP (S1 Fig) to see if the association between childhood SEP and physical capability in late-middle age exists independently of adult SEP [29] which would support the hypothesis that childhood is a sensitive period for socioeconomic disparities in relation to later life physical capability. The findings were compared to crude and cohort-adjusted analyses re-run on the same sample with complete information on all variables.

Data management was performed using SAS software, version 9.4 for Windows, and statistical analyses were conducted using the statistical software R version 3.2.2.

## Results

In the MC, participants were most often born to a father of occupational class two-five, and in the CPC, the majority belonged to the low social class in childhood. The majority of the study population was born to a married mother, especially in the MC (Table 1). Overall, the physical capability test scores of the MC appeared to be slightly poorer than those of the male participants in the CPC, and MC participants were slightly older than CPC participants (Table 1).

**Table 1:** Characteristics of the study population according to birth cohort and sex.

		The Metropolit Cohort		The Copenhagen Perinatal Cohort			
		Men		Men		Women	
		N/median	(%/IQR)	N/median	(%/IQR)	N/median	(%/IQR)
Total		2,486		752		966	
Age at CAMB participation, years							
	Median, IQR	56.7	56.3, 57.0	50.1	49.6, 50.6	50.1	49.6, 50.6
Father's occupational class							
	1	185	8.2				
	2	452	20				
	3	582	25.7				
	4	534	23.6				
	5	510	22.5				
	missing	223					
Parental social class							
	High			185	29.4	235	29
	Medium			203	32.3	246	30.4
	Low			241	38.3	329	40.6
	missing			123	-	156	-
Maternal marital status							
	Married	2,258	94.6	523	70.2	642	66.9
	Unmarried	130	5.4	222	29.8	318	33.1
	missing	98		7	-	6	-
Physical capability in late-middle age							
	Balance, cm^2^	906.0	701.0, 1,178.2	819.0	632.0, 1074.0	672.0	530.8, 874.0
	missing	126		13	-	22	-
	Flexibility, cm	7.0	0.0, 15.0	7.0	-1.0, 14.0	-2.0	-8.0, 4.0
	missing	305		63	-	126	-
	Jump height, cm	21.2	18.0, 24.2	23.7	20.6, 27.0	14.8	12.3, 17.4
	missing	349		88	-	161	-
	Lower back force, Newton	167.1	138.5, 198.9	172.1	144.1, 209.7	97.3	76.0, 119.8
	missing	520		117	-	129	-
	Abdominal force, Newton	168.7	142.4, 200.8	187.2	157.5, 217.5	104.4	85.5, 125.4
	missing	498		111	-	121	-
	Grip strength, kg	49.0	43.7, 54.6	51.1	45.2, 56.5	31.9	28.6, 35.2
	missing	9		<5	-	<5	-
	Chair rise, counts in 30 seconds	21.3	17.7, 25.3	23.2	19.3, 27.8	21.4	17.5, 25.7
	missing	314		63	-	57	-

The Copenhagen Aging and Midlife Biobank 2009–2011, Denmark. IQR: Inter-quartile range

**Fig 2:**
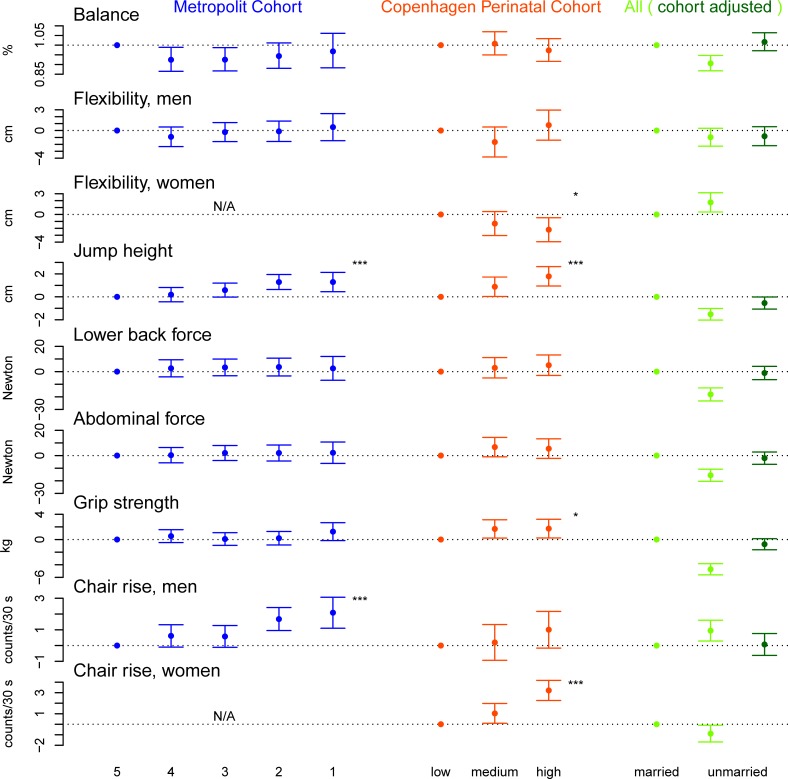
Results from linear regression analyses of the association between childhood socioeconomic position and physical capability in late-middle age. Childhood Socioeconomic Position is Represented by Father’s Occupational Class for the Metropolit Cohort, Parental Social Class for the Copenhagen Perinatal Cohort and Maternal Marital Status for Both Cohorts. Results are Presented According to Sex where Statistically Significant Interactions were Observed (Only Men were Included in the Metropolit Cohort). For Father’s Occupational Class and Parental Social Class, Tests for Trend were Run (Two-Sided P values: *** P < 0.001, ** P < 0.01, * P < 0.05).

Fig 2 shows the main results for the association between childhood SEP and physical capability in late-middle age. The most consistent associations were observed in relation to jump height. Being born to a father of higher occupational class or higher social class at birth was associated with higher jump height for occupational classes four-one, respectively: (0.19 cm (95% CI: -0.44, 0.82), 0.59 cm (95% CI: -0.02, 1.19), 1.29 cm (95% CI: 0.64, 1.94), 1.29 cm (95% CI: 0.45, 2.13); and for medium and high social class, respectively: 0.88 cm (95% CI: 0.03, 1.72) and 1.79 cm (95% CI: 0.94, 2.63)). Likewise, being born to an unwed mother was associated with poorer jump height (crude: -1.52 cm (95% CI: -2.02, -1.02), adjusted for birth cohort -0.54 cm (95% CI: -1.07, -0.01)). Higher parental social class at birth was also associated with better scores on grip strength for medium and high social class, respectively: (1.68 kg (95% CI: 0.23, 3.14) and 1.73 kg (95% CI: 0.25, 3.21)).

Analyses of the associations between maternal marital status and physical capability measures in late-middle age were similar in the two birth cohorts and no statistically significant interactions were observed. Analyses stratified according to year of birth did not reveal any differences (results not presented). From analyses corrected for anticipated selection bias we found that this bias most likely has led to underestimation of the associations between childhood SEP and physical capability in late-middle age. The bias analyses were conducted using the binary exposure maternal marital status as indicator of SEP together with binary outcome variables (<median vs. ≥median). These showed 53–66%-points increase in relative risks for physical capability below the median (depending on outcome measure used) associated with a hypothesized 10%-points lower participation among poor functioning individuals. Details regarding these analyses are provided in S1 File.

Sex stratified analyses performed on the CPC and the full sample showed that childhood SEP measures related differently to flexibility and chair rise performance according to sex (Fig 2). Among men, no association between parental social class and flexibility was observed, while for women, being born in higher social class or being born to a married mother was associated with poorer flexibility (-1.31 cm (95% CI: -3.05, 0.42) and -2.20 cm (95% CI: -3.94, -0.47) for medium and high social class, respectively). For men, being born to a father of higher occupational class was associated with better chair rise performance (0.61 counts/30 s (95% CI: -0.10, 1.32), 0.58 counts/30 s (95% CI: -0.11, 1.27), 1.68 counts/30 s (95% CI: 0.95, 2.41), and 2.08 counts/30 s (95% CI: 1.10, 3.07) for occupational class four-one, respectively), but no significant association was present in relation to parental social class or maternal marital status (after adjustment for birth cohort). For women, being born in higher social class was associated with better chair rise performance (1.04 counts/30 s (95% CI: 0.09, 1.98), and 3.22 counts/30 s (95% CI: 2.26, 4.18) for medium and high social class, respectively) and being born to an unwed mother was associated with poorer performance (-0.89 counts/30 s (95% CI: -0.69, -0.10)).

Adjustment for intermediate variable adult SEP (S1 Fig) attenuated the associations with jump height and chair rise performance. For flexibility, no change was observed among men, while for women, the previously observed relationship of higher childhood SEP being associated with poorer score was attenuated (S1–S3 Tables).

## Discussion

In this study, higher childhood SEP was associated with better jump height in late-middle age. Further, higher childhood SEP was related to better chair rise performance and grip strength, while among women higher childhood SEP was associated with reduced flexibility. Utilization of different indicators of childhood SEP unveiled minor differences in these estimates and for maternal marital status, the associations were statistically insignificant. As expected, adjustment for adult SEP attenuated most of the observed associations, indicating that childhood SEP is working through this pathway. For jump height and chair rise performance, the statistically significant associations persisted after adjustment for adult SEP.

A meta-analysis from 2011 reported higher childhood SEP to be associated with better adult grip strength [4]. The results are supported by a recent Swedish study [30] but conflict with findings from an Israeli cohort (adjusted for current household income) where the association was stronger and statistically significant for only women [12]. In accordance with the meta-analysis we did not detect any sex differences and adjustment for adult SEP attenuated our estimates [4].

Previous findings suggest that higher childhood SEP is associated with shorter time to complete five chair rises in adult life [4]. Direct comparison with our findings is problematic because we used a measure of maximum number of chair rises in 30 seconds. However, overall the evidence suggests that higher childhood SEP is related to more favorable chair rise performance. Adjustment for adult SEP did not fully explain the relationship in previous or our studies [4,12].

Finally, previous studies found that higher childhood SEP was associated with better odds of being able to balance for five seconds. The relationship was not statistically significant for all SEP indicators and adjustment for adult SEP led to no association [4]. Our results yielded no association when using postural sway area during 30 seconds as a balance measure and adjustment for adult SEP did not change this. Explanations for the deviating findings could include limitations related to the balance measure used and selection bias.

The strenuous measure of jump height appeared to better discriminate between levels of physical capability in this population in late-middle age compared to more gentle tests (e.g. balance). A similar tendency seemed to apply to the results for chair rise performance, which also requires the ability to lift your body weight explosively and involves many of the same muscles [26,31]. Considering that the study participants constitute a healthy group still active in the labor market, it is not surprising that only the more strenuous measures appear to discriminate the stronger from the weaker participants. The associations persisted for both jump height and chair rise performance even after adjustment for adult SEP. This could indicate that lower childhood SEP negatively impacts the physiological reserve in young adulthood (Fig 1) for example via health behavior, health and physiological development at a young age (S1 Fig), and that adult SEP cannot fully counteract this. The more gentle measures of physical capability are likely to become increasingly relevant as the cohort ages and the participants weaken. Measures of balance, lower back force, abdominal force and flexibility might have potential to shed light on SEP differences in for example falls and abilities to get out of bed and get dressed in older age. Thus, we find it relevant to explore SEP differences in such outcomes in older age.

Overall, the relationships between childhood SEP and physical capability in late-middle age were comparable among male and female participants in our study. The inconsistencies between previous studies could partly be due to between country differences in the extent to which women are active in the labor market. One could argue that the pathway through which childhood SEP relates to physical capability in late-middle age is different among women who are not active in the labor market because their subsequent SEP and lifestyle might depend more on the SEP of their husbands (if any). Our findings did suggest one interesting interaction. Higher childhood SEP was related to reduced flexibility in women, but not in men. We have no previous evidence to relate this to, but an explanation could include differential engagement in physical exercise in different SEP groups, which is more pronounced among women. Women of higher childhood SEP tend to engage more in leisure-time physical activity throughout life compared to those of low SEP [32]. Additionally, recent cross-sectional findings in late-middle age from the CAMB cohort have shown that high SEP participants engaged more in leisure-time physical activities of greater intensity and shorter duration compared to their low SEP counterparts [33], which may reduce sagittal flexibility. From a prevention perspective, it is relevant to investigate when sex differences in SEP-capability arises, as previous cross-sectional findings from the CAMB have not found an interaction between occupational social class in late-middle age and sex in relation to physical capability [25]. This suggests that women may be more sensitive to early life SEP in relation to later flexibility when compared to men.

Adjustment for adult SEP generally attenuated, but did not fully explain the association between childhood SEP and physical capability in late-middle age. This is in line with previous findings [6,34,35] and supports a proposed hypothesis of childhood being important to later life physical health, regardless of adult life [6]. Arguments include negative impacts on physiological reserve development [36] for example via birth size, gestational age or growth restriction in utero [22,37,38]. However, because we only have a single measure of adult SEP we cannot address the differential effects of SEP across adulthood on the association of interest [39].

Our study found minor variations in results according to SEP indicator, supporting the hypothesis that differences in the association between childhood SEP and later life physical capability could be due to the use of different SEP indicators [4]. Parental SEP and father’s occupational class were most strongly associated with physical capability. Parental SEP included a number of aspects (breadwinner’s education, occupation, income type, and housing quality) that arguably are relevant to health during childhood [40]. It is, however, questionable whether all aspects are equally important, which is assumed when constructing an unweighted sum variable. Father’s occupational class is a widely used measure in European studies, which makes comparisons across studies easy. However, this measure is not relevant to single-mother households and fails to capture other aspects of SEP. Maternal marital status was generally not associated with physical capability in late-middle age in our study. This may reflect that this exposure measure is a crude and poor proxy for childhood SEP. If for example, a large proportion of the unmarried mothers got married shortly after giving birth, this could affect the results similarly to non-differential misclassification with an underestimation of the true association because the marital status was not subsequently reclassified.

The strengths of this study include its longitudinal design with lifelong follow-up, the prospectively collected measures and the detailed objectively measured outcomes including additional aspects of physical capability compared to previous studies. To our knowledge, this is also the first study using such data to address the question of whether childhood SEP bear into physical capability in late-middle age in a Nordic welfare state, an association that has repeatedly been demonstrated in mainly British data.

Our findings should be interpreted bearing the limitations in mind. Only 32% of the invited MC and CPC members participated in the CAMB follow-up. A previous comparison of CAMB participants with non-participants using Danish national registries showed that participants were significantly less likely to die in the years following data collection than the non-participants, had higher educational attainment and were more active in the labor market [18]. In the quantitative bias analyses, we found that the selection most likely results in an underestimation of the associations. Also, we were unable to adjust for unmeasured confounders such as parental intelligence and parental chronic hereditary disease. Adjustment for variables later on the pathway e.g. participants’ intelligence or health was not an alternative because it would introduce collider stratification bias. This uncontrolled confounding could have biased our results towards no association. However, since the biases are likely to be towards null, we find it unlikely that they can explain the positive findings. Finally, adjustment for adult SEP using highest attained educational level may result in residual confounding as this measure might not capture the full spectrum of SEP. Such single-point-in-time measure can mask large life course variation, although educational level is relatively stable after young adulthood and is related to health [40].

The collective evidence from this and previous studies adds support to the effectiveness of prevention strategies focusing on early life social determinants of later physical capability. The Organisation for Economic Co-operation and Development and the World Health Organization describe in more general terms how interventions targeting early child development and early school education stand as cost effective ways of promoting better health. The early interventions appear to be more cost effective than those in later life [41]. Policies focusing on lifting children out of low SEP at an early age appear to be the most effective from a prevention perspective.

In conclusion, this study lends some support to the previously suggested modest relationship between childhood SEP and physical capability in late-middle age, mainly for the more strenuous jump height and chair rise performance tests.

## Supporting information

S1 Fig Directed acyclic graph. The hypothesized relationship between childhood socioeconomic position (SEP) and physical capability in late-middle age.
(TIF)

S1 Table Fathers occupational class adjusted for adult socioeconomic position (SEP). Crude and Adult Socioeconomic Position Adjusted Results from Linear Regression Analyses of the Association between Father’s Occupational Class and Physical Capability Measures in Late-Middle Age. Participants from the Metropolit Cohort (Boys Born in 1953) who Participated in the Copenhagen Aging and Midlife Biobank 2009–2011, Denmark.
(DOCX)

S2 Table Parental social class adjusted for adult socioeconomic position (SEP). Crude and Adult Socioeconomic Position Adjusted Results from Linear Regression Analyses of the Association between Parental Social Class and Physical Capability Measures in Late-Middle Age Presented According to Sex where Statistically Significant Interactions were Observed. Participants from the Copenhagen Perinatal Cohort (Boys and Girls Born in 1959–1961) who Participated in the Copenhagen Aging and Midlife Biobank 2009–2011, Denmark.
(DOCX)

S3 Table Maternal marital status adjusted for adult socioeconomic position (SEP). Crude and Adjusted Results from Linear Regression Analyses of the Association between Maternal Marital Status and Physical Capability Measures in Late-Middle Age Presented According to Sex where Statistically Significant Interactions were Observed. Participants from the Metropolit Cohort (Boys born in 1953) and Copenhagen Perinatal Cohort (Boys and Girls Born in 1959–1961) who Participated in the Copenhagen Aging and Midlife Biobank 2009–2011, Denmark.
(DOCX)

S1 File Simple quantitative selection bias analysis. (DOCX)

## References

[1] Turrell G, Lynch JW, Leite C, Raghunathan T, Kaplan GA. Socioeconomic disadvantage in childhood and across the life course and all-cause mortality and physical function in adulthood: Evidence from the Alameda County Study. J Epidemiol Community Health. 2007;61: 723–30. 10.1136/jech.2006.050609 17630374 PMC2653004

[2] Haas S. Trajectories of functional health: The “long arm” of childhood health and socioeconomic factors. Soc Sci Med. 2008;66: 849–861. 10.1016/j.socscimed.2007.11.004 18158208

[3] Laaksonen E, Martikainen P, Head J, Rahkonen O, Marmot MG, Lahelma E. Associations of multiple socio-economic circumstances with physical functioning among Finnish and British employees. Eur J Public Health. 2009;19: 38–45. 10.1093/eurpub/ckn123 19060329 PMC2639014

[4] Birnie K, Cooper R, Martin RM, Kuh D, Sayer AA, Alvarado BE, et al Childhood socioeconomic position and objectively measured physical capability levels in adulthood: a systematic review and meta-analysis. PLoS.One. 2011 p. e15564–. 10.1371/journal.pone.0015564 21297868 PMC3027621

[5] Strand BH, Cooper R, Hardy R, Kuh D, Guralnik J. Lifelong socioeconomic position and physical performance in midlife: results from the British 1946 birth cohort. Eur J Epidemiol. 2011;26: 475–483. 10.1007/s10654-011-9562-9 21416275 PMC3246593

[6] Hurst L, Stafford M, Cooper R, Hardy R, Richards M, Kuh D. Lifetime socioeconomic inequalities in physical and cognitive aging. Am J Public Health. 2013;103: 1641–1648. 10.2105/AJPH.2013.301240 23865666 PMC3780680

[7] Ploubidis GB, Benova L, Grundy E, Laydon D, DeStavola B. Lifelong Socio Economic Position and biomarkers of later life health: Testing the contribution of competing hypotheses. Soc Sci Med. Elsevier Ltd; 2014;119: 258–265. 10.1016/j.socscimed.2014.02.018 24636422

[8] Mishra GD, Black S, Stafford M, Cooper R, Kuh D. Childhood and Maternal Effects on Physical Health Related Quality of Life Five Decades Later: The British 1946 Birth Cohort. PLoS One. 2014;9: 1–9. 10.1371/journal.pone.0088524 24670776 PMC3966737

[9] Sousa ACP de A, Guerra RO, Tu MT, Philips SP, Guralnik JM, Zunzunegui M-V. Lifecourse Adversity and Physical Performance across Countries among Men and Women Aged 65–74. PLoS One. 2014;9: 1–10. 10.1371/journal.pone.0102299 25101981 PMC4125146

[10] Agahi N, Shaw BA, Fors S. Social and economic conditions in childhood and the progression of functional health problems from midlife into old age. J Epidemiol Community Heal. 2014;68: 734–740. 10.1136/jech-2013-203698 24759781 PMC4112427

[11] Henretta JC, McCrory C. Childhood Circumstances and Mid-Life Functional Mobility. J Aging Heal. 2015;28: 440–459. 10.1177/0898264315594135 26148942

[12] Weinstein G. Childhood conditions and current physical performance among non-institutionalized individuals aged 50+ in Israel. Eur J Ageing. 2016;13: 335–347. 10.1007/s10433-016-0380-5 28190995 PMC5300071

[13] Mollborn S, Lawrence E, James-Hawkins L, Fomby P. When do socioeconomic resources matter most in early childhood? Adv Life Course Res. Elsevier Ltd; 2014;20: 56–69. 10.1016/j.alcr.2014.03.001 25431546 PMC4242154

[14] Ben-shlomo Y, Kuh D. A life course approach to chronic disease epidemiology: conceptual models, empirical challenges and interdisciplinary perspectives. Int J Epidemiol. 2002;31: 285–293. 11980781

[15] Strachan DP, Sheikh A. A life course approach to respiratory and allergic diseases In: Kuh D, Ben-Shlomo Y, editors. A life course approach to chronic disease epidemiology. 2nd ed 10: Oxford University Press; 2004 pp. 240–259.

[16] Lara J, Cooper R, Nissan J, Ginty AT, Khaw K-T, Deary IJ, et al A proposed panel of biomarkers of healthy ageing. BMC Med. BMC Medicine; 2015;13: 222 10.1186/s12916-015-0470-9 26373927 PMC4572626

[17] Cooper R, Kuh D, Hardy R, Mortality Review Group, FALCon and HALCyon Study Teams. Objectively measured physical capability levels and mortality: systematic review and meta-analysis. BMJ. 2010;341: c4467 10.1136/bmj.c4467 20829298 PMC2938886

[18] Lund R, Mortensen EL, Christensen U, Bruunsgaard H, Holm-Pedersen P, Fiehn N-E, et al Cohort Profile: The Copenhagen Aging and Midlife Biobank (CAMB). Int J Epidemiol. 2016;45: 1044–1053. 10.1093/ije/dyv149 26210613

[19] Osler M, Lund R, Kriegbaum M, Christensen U, Andersen AMN. Cohort profile: The Metropolit 1953 Danish male birth cohort. Int J Epidemiol. 2006;35: 541–545. 10.1093/ije/dyi300 16377658

[20] Zachau-Christiansen B. Development during the first year of life [Internet]. Helsingør: Poul Andersens Forlag; 1972 Available: http://books.google.dk/books/about/Development_during_the_first_year_of_lif.html?id=2GU0ywAACAAJ&pgis=1

[21] Weston R, Smyth B. Financial living standards after divorce. Fam Matters. 2000; 10–15.

[22] Pattenden S, Dolk H, Vrijheid M. Inequalities in low birth weight: parental social class, area deprivation, and “lone mother” status. J Epidemiol Community Health. 1999;53: 355–8. 10.1136/jech.53.6.355 10396482 PMC1756885

[23] Modin B. Born out of wedlock and never married—It breaks a man’s heart. Soc Sci Med. 2003;57: 487–501. 10.1016/S0277-9536(02)00374-X 12791491

[24] Avlund K, Osler M, Mortensen EL, Christensen U, Bruunsgaard H, Holm-Pedersen P, et al Copenhagen Aging and Midlife Biobank (CAMB): An Introduction. J Aging Health. 2014;26: 5–20. 10.1177/0898264313509277 24584257

[25] Hansen AM, Andersen LL, Skotte J, Christensen U, Mortensen OS, Molbo D, et al Social Class Differences in Physical Functions in Middle-Aged Men and Women. J Aging Health. 2014;26: 88–105. 10.1177/0898264313508188 24584262

[26] Holsgaard Larsen A, Caserotti P, Puggaard L, Aagaard P. Reproducibility and relationship of single-joint strength vs multi-joint strength and power in aging individuals. Scand J Med Sci Sport. 2007;17: 43–53. 10.1111/j.1600-0838.2006.00560.x 16787447

[27] FAQ How do I interpret a regression model when some variables are log transformed? In: UCLA Institute for Digital Research and Education [Internet]. 2016 [cited 1 Jun 2018]. Available: https://stats.idre.ucla.edu/sas/faq/how-can-i-interpret-log-transformed-variables-in-terms-of-percent-change-in-linear-regression/

[28] Lash T, Fox M, Fink A. Applying quantitative bias analysis to epidemiologic data Springer Science & Business Media; 2011.

[29] Hertzman C, Power C. Health and Human Development: Understandings From Life-Course Research. Dev Neuropsychol. 2003;24: 719–744. 10.1080/87565641.2003.9651917 14561568

[30] Sternäng O, Reynolds C a., Finkel D, Ernsth-Bravell M, Pedersen NL, Dahl Aslan a. K. Factors associated with grip strength decline in older adults. Age Ageing. 2014; 269–274. 10.1093/ageing/afu170 25362503 PMC4400526

[31] Ritchie C, Trost SG, Brown W, Armit C. Reliability and validity of physical fitness field tests for adults aged 55 to 70 years. J Sci Med Sport. 2005;8: 61–70. 10.1016/S1440-2440(05)80025-8 15887902

[32] Juneau CE, Benmarhnia T, Poulin AA, Côté S, Potvin L. Socioeconomic position during childhood and physical activity during adulthood: a systematic review. Int J Public Heal. 2015;60: 799–813. 10.1007/s00038-015-0710-y 26298440

[33] Petersen GL, Mortensen EL, Rod NH, Lange T, Flensborg-Madsen T, Hansen ÅM, et al Occupational social class and personality traits in relation to leisure-time physical activity level: cross-sectional results from the Copenhagen Aging and Midlife Biobank. J Aging Health. 2018;30: 1263–1283. 10.1177/0898264317714928 28752788

[34] Osler M, Madsen M, Nybo Andersen AM, Avlund K, Mcgue M, Jeune B, et al Do childhood and adult socioeconomic circumstances influence health and physical function in middle-age? Soc Sci Med. Elsevier Ltd; 2009;68: 1425–1431. 10.1016/j.socscimed.2009.01.014 19272688 PMC2690645

[35] Birnie K, Martin RM, Gallacher J, Bayer A, Gunnell D, Ebrahim S, et al Socio-economic disadvantage from childhood to adulthood and locomotor function in old age: a lifecourse analysis of the Boyd Orr and Caerphilly prospective studies. J Epidemiol.Community Health. 2010 pp. 1014–1023. 10.1136/jech.2009.103648 20644236 PMC3381706

[36] Ferrucci L, Cooper R, Shardell M, Simonsick EM, Schrack JA, Kuh D. Age-related change in mobility: Perspectives from life course epidemiology and geroscience. Journals Gerontol—Ser A Biol Sci Med Sci. 2016;71: 1184–1194. 10.1093/gerona/glw043 26975983 PMC4978365

[37] Beard JR, Lincoln D, Donoghue D, Taylor D, Summerhayes R, Dunn TM, et al Socioeconomic and maternal determinants of small-for-gestational age births: patterns of increasing disparity. Acta Obstet Gynecol Scand. 2009;88: 575–83. 10.1080/00016340902818170 19330564

[38] Blumenshine P, Egerter S, Barclay CJ, Cubbin C, Braveman PA. Socioeconomic disparities in adverse birth outcomes: A systematic review. Am J Prev Med. Elsevier Inc.; 2010;39: 263–272. 10.1016/j.amepre.2010.05.012 20709259

[39] House JS, Lantz PM, Herd P. Continuity and change in the social stratification of aging and health over the life course: evidence from a nationally representative longitudinal study from 1986 to 2001/2002 (Americans’ Changing Lives Study). J Gerontol B Psychol Sci Soc Sci. 2005;60B: 15–26. 10.1093/geronb/60.Special_Issue_2.S15 16251586

[40] Galobardes B, Shaw M, Lawlor D a, Lynch JW, Davey Smith G. Indicators of socioeconomic position (part 1). J Epidemiol Community Health. 2006;60: 7–12. 10.1136/jech.2004.023531 16361448 PMC2465546

[41] OECD/WHO. Promoting Health, Preventing Disease. The economic case [Internet]. 2015. 10.1097/00017285-198805000-00004

