# Correction: Childhood socioeconomic position and physical capability in late-middle age in two birth cohorts from the Copenhagen aging and midlife biobank

**DOI:** 10.1371/journal.pone.0210199

**Published:** 2018-12-28

**Authors:** Gitte Lindved Petersen, Jolene Lee Masters Pedersen, Naja Hulvej Rod, Erik Lykke Mortensen, Ichiro Kawachi, Merete Osler, Åse Marie Hansen, Rikke Lund

The physical capability measure of flexibility was interpreted incorrectly in the article. Negative values represent greater flexibility, meaning that the participant was able to push finger tips below the feet when standing on a box with stretched legs. The sizes of the estimates are correct, but they should be interpreted as lower values being more favorable, and not oppositely as described in the article.
