# Supplementary figures and images for: Childhood socioeconomic position and physical capability in late-middle age in two birth cohorts from the Copenhagen aging and midlife biobank
Source: PLoS One. 2018 Oct 1;13(10):e0205019. doi: 10.1371/journal.pone.0205019 (PMC6166988; doi:10.1371/journal.pone.0205019)

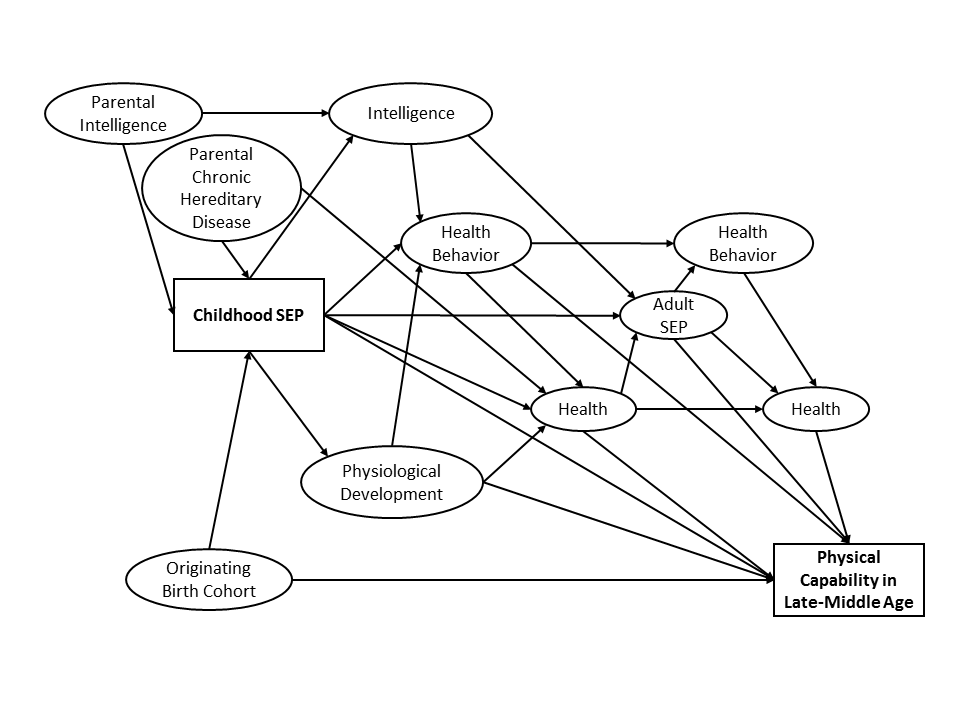

Supplement: S1 Fig — The hypothesized relationship between childhood socioeconomic position (SEP) and physical capability in late-middle age. (TIF) [file pone.0205019.s001.tif]
