# Supplementary material for: Childhood socioeconomic position and physical capability in late-middle age in two birth cohorts from the Copenhagen aging and midlife biobank
Source: PLoS One. 2018 Oct 1;13(10):e0205019. doi: 10.1371/journal.pone.0205019 (PMC6166988; doi:10.1371/journal.pone.0205019)
Supplement: S1 Table — Crude and Adult Socioeconomic Position Adjusted Results from Linear Regression Analyses of the Association between Father’s Occupational Class and Physical Capability Measures in Late-Middle Age. Participants from the Metropolit Cohort (Boys Born in 1953) who Participated in the Copenhagen Aging and Midlife Biobank 2009–2011, Denmark. (DOCX) [file pone.0205019.s002.docx]

# Supporting information

S1 Table**. Fathers occupational class adjusted for adult socioeconomic position (SEP).** Crude and Adult Socioeconomic Position Adjusted Results from Linear Regression Analyses of the Association between Father’s Occupational Class and Physical Capability Measures in Late-Middle Age. Participants from the Metropolit Cohort (Boys Born in 1953) who Participated in the Copenhagen Aging and Midlife Biobank 2009-2011, Denmark.

|  |  |  | Crude | | Adjusted for adult SEP | |
| --- | --- | --- | --- | --- | --- | --- |
|  |  | N | Β^a^ | 95% CI | β ^a^ | 95% CI |
| Balance, cm² | 5 | 477 | 1 | reference | 1 | reference |
|  | 4 | 499 | 0.93 | 0.87, 0.99 | 0.93 | 0.87, 1.00 |
|  | 3 | 557 | 0.92 | 0.87, 0.99 | 0.94 | 0.88, 1.01 |
|  | 2 | 433 | 0.94 | 0.88, 1.01 | 0.97 | 0.90, 1.04 |
|  | 1 | 177 | 0.97 | 0.88, 1.06 | 1.00 | 0.91, 1.10 |
| Flexibility, cm | 5 | 438 | 0 | reference | 0 | reference |
|  | 4 | 467 | -0.86 | -2.28, 0.56 | -0.80 | -2.22, 0.63 |
|  | 3 | 524 | -0.19 | -1.57, 1.19 | -0.17 | -1.58, 1.25 |
|  | 2 | 393 | -0.10 | -1.59, 1.38 | -0.09 | -1.62, 1.45 |
|  | 1 | 162 | 0.57 | -1.39, 2.53 | 0.43 | -1.64, 2.51 |
| Jump height, cm | 5 | 428 | 0 | reference | 0 | reference |
|  | 4 | 453 | 0.19 | -0.43, 0.82 | 0.00 | -0.62, 0.62 |
|  | 3 | 510 | 0.51 | -0.09, 1.12 | 0.08 | -0.53, 0.69 |
|  | 2 | 384 | 1.27 | 0.62, 1.92 | 0.70 | 0.04, 1.37 |
|  | 1 | 168 | 1.30 | 0.46, 2.14 | 0.44 | -0.44, 1.32 |
| Lower back force, Newton | 5 | 392 | 0 | reference | 0 | reference |
|  | 4 | 417 | 2.68 | -4.12, 9.49 | 1.97 | -4.86, 8.80 |
|  | 3 | 471 | 3.08 | -3.54, 9.69 | 2.09 | -4.65, 8.82 |
|  | 2 | 360 | 3.28 | -3.79, 10.34 | 1.58 | -5.72, 8.88 |
|  | 1 | 142 | 2.99 | -6.49, 12.47 | 1.00 | -9.00, 10.99 |
| Abdominal force, Newton | 5 | 397 | 0 | reference | 0 | reference |
|  | 4 | 425 | 0.50 | -5.57, 6.56 | -0.18 | -6.26, 5.91 |
|  | 3 | 473 | 1.82 | -4.10, 7.73 | 1.01 | -5.01, 7.03 |
|  | 2 | 364 | 1.82 | -4.49, 8.13 | 0.75 | -5.77, 7.27 |
|  | 1 | 143 | 3.03 | -5.45, 11.51 | 2.06 | -6.88, 11.00 |
| Grip strength, kg | 5 | 506 | 0 | reference | 0 | reference |
|  | 4 | 533 | 0.57 | -0.46, 1.59 | 0.42 | -0.61, 1.45 |
|  | 3 | 575 | 0.09 | -0.92, 1.10 | -0.19 | -1.22, 0.84 |
|  | 2 | 449 | 0.16 | -0.91, 1.23 | -0.20 | -1.31, 0.91 |
|  | 1 | 184 | 1.33 | -0.09, 2.76 | 0.80 | -0.71, 2.31 |
| Chair rise, counts in 30 seconds | 5 | 432 | 0 | reference | 0 | reference |
|  | 4 | 459 | 0.62 | -0.09, 1.33 | 0.37 | -0.33, 1.07 |
|  | 3 | 511 | 0.55 | -0.15, 1.24 | -0.01 | -0.70, 0.69 |
|  | 2 | 405 | 1.69 | 0.96, 2.42 | 0.90 | 0.16, 1.65 |
|  | 1 | 157 | 2.05 | 1.06, 3.03 | 0.81 | -0.21, 1.84 |

CI: Confidence Interval; SEP: Socioeconomic position; ^a^β for balance is interpreted as relative change.
