# Supplementary material for: Childhood socioeconomic position and physical capability in late-middle age in two birth cohorts from the Copenhagen aging and midlife biobank
Source: PLoS One. 2018 Oct 1;13(10):e0205019. doi: 10.1371/journal.pone.0205019 (PMC6166988; doi:10.1371/journal.pone.0205019)
Supplement: S3 Table — Crude and Adjusted Results from Linear Regression Analyses of the Association between Maternal Marital Status and Physical Capability Measures in Late-Middle Age Presented According to Sex where Statistically Significant Interactions were Observed. Participants from the Metropolit Cohort (Boys born in 1953) and Copenhagen Perinatal Cohort (Boys and Girls Born in 1959–1961) who Participated in the Copenhagen Aging and Midlife Biobank 2009–2011, Denmark. (DOCX) [file pone.0205019.s004.docx]

# Supporting information

**S3 Table.** Maternal marital status adjusted for adult socioeconomic position (SEP). Crude and Adjusted Results from Linear Regression Analyses of the Association between Maternal Marital Status and Physical Capability Measures in Late-Middle Age Presented According to Sex where Statistically Significant Interactions were Observed. Participants from the Metropolit Cohort (Boys born in 1953) and Copenhagen Perinatal Cohort (Boys and Girls Born in 1959-1961) who Participated in the Copenhagen Aging and Midlife Biobank 2009-2011, Denmark.

|  | |  | Crude | | Adjusted for cohort | | Adjusted for cohort and adult SEP | |
| --- | --- | --- | --- | --- | --- | --- | --- | --- |
|  | | N | β^a^ | 95% CI | β^a^ | 95% CI | β^a^ | 95% CI |
| Balance, cm² | Married | 3282 | 1 | reference | 1 | reference | 1 | reference |
|  | Unmarried | 645 | 0.91 | 0.87, 0.95 | 1.02 | 0.97, 1.07 | 1.00 | 0.96, 1.05 |
| Flexibility, cm (men only) | Married | 2459 | 0 | reference | 0 | reference | 0 | reference |
|  | Unmarried | 305 | -0.97 | -2.26, 0.33 | -0.81 | -2.19, 0.57 | -0.89 | -2.28, 0.50 |
| Flexibility, cm (women only) | Married | 566 | 0 | reference | 0 | reference | 0 | Reference |
|  | Unmarried | 268 | 1.75 | 0.36, 3.14 | 1.75 | 0.36, 3.14 | 1.39 | -0.06, 2.84 |
| Jump height, cm | Married | 2949 | 0 | reference | 0 | reference | 0 | reference |
|  | Unmarried | 546 | -1.52 | -2.02, -1.01 | -0.54 | -1.07, -0.02 | -0.22 | -0.75, 0.31 |
| Lower back force, Newton | Married | 2788 | 0 | reference | 0 | reference | 0 | reference |
|  | Unmarried | 545 | -18.11 | -23.31, -12.92 | -1.13 | -6.39, 4.13 | -1.15 | -6.48, 4.17 |
| Abdominal force, Newton | Married | 2817 | 0 | reference | 0 | reference | 0 | reference |
|  | Unmarried | 551 | -15.47 | -20.22, -10.71 | -1.94 | -6.83, 2.95 | -2.37 | -7.32, 2.58 |
| Grip strength, kg | Married | 3401 | 0 | reference | 0 | reference | 0 | reference |
|  | Unmarried | 667 | -4.72 | -5.61, -3.83 | -0.75 | -1.63, 0.14 | -0.69 | -1.58, 0.20 |
| Chair rise, counts in 30 seconds (men only) | Married | 2441 | 0 | reference | 0 | reference | 0 | reference |
|  | Unmarried | 314 | 0.94 | 0.27, 1.60 | 0.04 | -0.66, 0.73 | 0.49 | -0.20, 1.18 |
| Chair rise, counts in 30 seconds (women only) | Married | 612 | 0 | reference | 0 | reference | 0 | reference |
|  | Unmarried | 291 | -0.89 | -1.69, -0.10 | -0.89 | -1.69, -0.10 | -0.28 | -1.08, 0.52 |

## CI: Confidence Interval; SEP: Socioeconomic position; ^a^β for balance is interpreted as relative change.
