# Supplementary material for: Childhood socioeconomic position and physical capability in late-middle age in two birth cohorts from the Copenhagen aging and midlife biobank
Source: PLoS One. 2018 Oct 1;13(10):e0205019. doi: 10.1371/journal.pone.0205019 (PMC6166988; doi:10.1371/journal.pone.0205019)
Supplement: S1 File — (DOCX) [file pone.0205019.s005.docx]

# Supporting information

## S1 File. Simple quantitative selection bias analysis

To address the potential impact of selection bias, we conducted a simple quantitative bias analysis based on the methods by Lash *et al.* (1). Our study sample was affected by selective dropout because low socioeconomic position (SEP) individuals were less likely to participate in the Copenhagen Aging and Midlife Biobank (CAMB) follow-up (2) and because exclusion criteria prevented participants in poor health to participate in a number of the physical capability tests. Poor physical health could also in itself have led to non-participation for an unknown proportion of those invited to CAMB. The association between childhood SEP and physical capability in late-middle age is likely to be different in CAMB participants compared to non-participants, and conditioning on participation can introduce a spurious association, resulting in biased results.


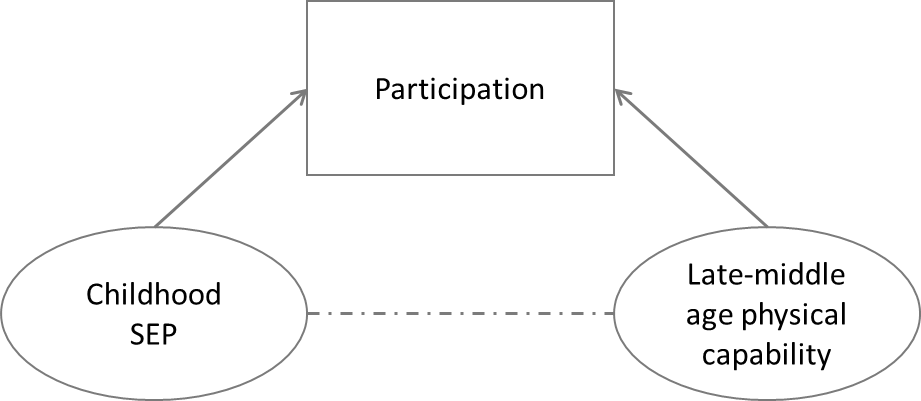


A previous comparison to National registers has shown that participants in the CAMB tests had a higher educational level compared to invited non-participants. Among non-respondents (N=10,746), 28.7% had a lower (primary or lower secondary) education, while this was 17.6% among those participating only in the CAMB questionnaire (N=7,191), and 15.4% among the CAMB test participants (N=5,576). On the other hand, 69.8% had higher (upper secondary to tertiary) educational level among the non-participants, 82.0% among questionnaire participants, and 84.3% among test participants (2). From this information we estimated the overall educational level as:

$$Estimated proportion in low SEP:\frac{0.287*10,746+0.176*7,191}{17,937}=0.24$$

$$Estimated proportion in high SEP:\frac{0.698*10,746+0.820*7191}{17,937}=0.75$$

For the bias analyses we created three binary outcome variables choosing a cut-point at the median score (<median vs. ≥median) as presented in Table 1. We chose jump height, grip strength, and lower back force because these represented respectively the outcomes with strongest/most consistent association with the exposure measures, highest participation, and lowest participation.

The figure below shows the selection bias adjusted relative risk (RR) estimates for different scenarios of assumed selection proportions in the low SEP/poor physical capability group. Since the estimated proportion of people in low SEP was 24%, the circled results can be seen as reference values with no correction for selection bias. Increasing selection (meaning lower participation) in the low SEP/poor physical capability group leads to increasing RR estimates with 10%-points lower participation leading to 53-66%-points higher RR.

It is not possible to estimate the selection proportion according to physical capability using register data. The above presented results lend support to the assumed underestimation of our findings if we anticipate that some degree of selective dropout exists in the low SEP group due to poor physical capability. It should, however, be noted that the selection bias adjusted results do not take potential confounding into account.

1. Lash T, Fox M, Fink A. Applying quantitative bias analysis to epidemiologic data. Springer Science & Business Media; 2011.

2. Lund R, Mortensen EL, Christensen U, Bruunsgaard H, Holm-Pedersen P, Fiehn N-E, et al. Cohort Profile: The Copenhagen Aging and Midlife Biobank (CAMB). Int J Epidemiol. 2016;45(4):1044–53.
